# The AMPK/GDF15 Axis: A Novel Target for the Neuroprotective Effects of Metformin in Ischemic Stroke

**DOI:** 10.1007/s12035-025-05126-7

**Published:** 2025-06-09

**Authors:** Ghadah H. Alshehri, Hayder M. Al-kuraishy, Bshra A. Alsfouk, Ali I. Al-Gareeb, Safaa A. Faheem, Athanasios Alexiou, Marios Papadakis, Gaber El-Saber Batiha

**Affiliations:** 1 Department of Pharmacy Practice, College of Pharmacy, Princess Nourah Bint Abdulrahman University, Riyadh, Saudi Arabia; 2 Department of Clinical Pharmacology and Medicine, College of Medicine, Mustansiriyah University, Baghdad, Iraq; 3 Department of Pharmaceutical Sciences, College of Pharmacy, Princess Nourah Bint Abdulrahman University, P.O. Box 84428, 11671 Riyadh, Saudi Arabia; 4 Department of Pharmacology and Toxicology, Faculty of Pharmacy, Egyptian Russian University, Cairo-Suez Road, Badr City, 11829 Egypt; 5 Department of Research & Development, Funogen, Attiki, 11741 Athens, Greece; 6 University Centre for Research & Development, Chandigarh University, Punjab Mohali, India; 7 University Hospital Witten-Herdecke, University of Witten-Herdecke, Heusnerstrasse 40, 42283 Wuppertal, Germany; 8 Department of Pharmacology and Therapeutics, Faculty of Veterinary Medicine, Damanhour University, AlBeheira, 22511 Egypt

**Keywords:** Metformin, AMP-activated protein kinase, Growth differentiation factor 15, Ischemic stroke

## Abstract

Metformin is an anti-diabetic drug used in the management of type 2 diabetes (T2D). Metformin has different pleiotropic effects, such as anti-inflammatory, antioxidant, antithrombotic, and vasculoprotective. Metformin has neuroprotective effects against neurodegenerative diseases and ischemic stroke. Conversely, metformin may exacerbate the pathogenesis of ischemic stroke. This controversial point may be related to the impact of metformin on the different signaling pathways, such as AMP-activated protein kinase (AMPK) and growth differentiation factor 15 (GDF-15). Many studies have reported the effect of metformin on ischemic stroke, with AMPK signaling only. However, little has been explored about the impact of metformin on the GDF-15 signaling in ischemic stroke. Accordingly, this review aims to discuss the role of metformin in the neuropathology of ischemic stroke regarding the AMPK and GDF-15 signaling pathways.

## Introduction

Metformin is a well-known anti-diabetic drug from the biguanide family used as a first-line in the management of type 2 diabetes (T2D) [1]. Metformin was first described in 1920, as it was derived from the *Galega officinalis* plant, which has been used widely in treating different diseases for over 4 centuries [2]. The hypoglycemic effect of metformin was initially reported by a French physician, Jean Stern, in 1957 [2]. In 1995, the FDA approved metformin for managing T2D [2]. In addition, metformin is commonly prescribed for treating other diseases, such as obesity, hepatic steatosis, and polycystic ovary syndrome [3].

It has been reported that prolonged use of metformin reduces mortality from cardiovascular complications through mechanisms independent of lowering blood glucose [4]. Metformin has different pleiotropic effects, such as anti-inflammatory, antioxidant, antithrombotic, and vasculoprotective effects [5–7]. Therefore, metformin was suggested to be effective in treating cardiovascular diseases such as atherosclerosis. Besides, many studies observed that metformin has neuroprotective effects against neurodegenerative diseases and ischemic stroke [8, 9]. However, other studies reported that metformin may exacerbate the pathogenesis of ischemic stroke [10]. This controversial point may be related to the effect of metformin on the different signaling pathways, such as AMP-activated protein kinase (AMPK) and growth differentiation factor 15 (GDF-15). Indeed, many studies indicated that the AMPK signaling pathway has dual neuroprotective and neuro-detrimental effects on the neuropathology of ischemic stroke [11]. Besides, GDF-15 serum level correlates with ischemic stroke severity [12]. Many studies have reported the effect of metformin on ischemic stroke through AMPK signaling. However, the effect of metformin on ischemic strokes related to GDF-15 signaling has been little explored. Accordingly, this review aims to discuss the potential role of metformin on the neuropathology of ischemic stroke regarding the AMPK and GDF-15 signaling pathway.

## Metformin Pharmacology

Metformin is an orally active drug, though it has low lipid solubility and is less absorbed from the intestines [13–15]. Thus, the bioavailability of metformin is about 50% of the administered dose [16]. Metformin requires specific organic cationic transporters (OCT) for its absorption from the intestines. Metformin absorption via OCT is a saturable process; therefore, there is an inverse association between the administered doses and the intestinal absorption rate [17–20]. Metformin is not metabolized and is mainly excreted by the kidney [21]. In addition, metformin has minimal plasma protein binding affinity. Thus, it has a short half-life of about 4–5 h [22]. Metformin is regarded as a safe drug and is less susceptible to drug-drug interaction [22]. However, prolonged and toxic doses of metformin may lead to the development of lactic acidosis, especially in patients with heart failure and renal impairment. Metformin can cross the blood–brain barrier (BBB) but needs time to produce the anorexigenic effect in diabetic patients [23].

The fundamental mechanism of metformin’s action is not fully clarified. However, the hypoglycemic effect of metformin is related to inhibiting gluconeogenesis, reducing intestinal absorption of glucose, and increasing peripheral insulin sensitivity [24]. The cellular effect of metformin is mediated by activating the expression and signaling pathways of AMPK, which is required for the inhibitory effect of metformin on hepatocyte production of glucose [24]. However, metformin can produce many pleiotropic and cellular impacts independent of the AMPK signaling pathway [25]. Metformin inhibits mitochondrial complex I, interfering with the mitochondrial respiratory chain, reducing cellular ATP, and increasing AMP with subsequent activation of AMPK [25].

Interestingly, AMPK activation reduces gluconeogenesis when the energy substrates are reduced [26, 27]. However, the neuronal store of nutrients is inattentive due to the absence of a glycolytic pathway. Therefore, a stressful condition such as ischemic stroke induces the over-activation of the AMPK signaling in the brain following ischemic stroke [28]. In addition, metformin inhibits mitochondrial glycerophosphate dehydrogenase, inhibiting hepatic gluconeogenesis [27]. Furthermore, metformin can bind copper ions, an essential metal for activating mitochondrial enzymes and ATP production [29]. Thus, the metal-binding effect of metformin could be an alternative mechanism for the action of metformin [30].

**Fig. 1:**
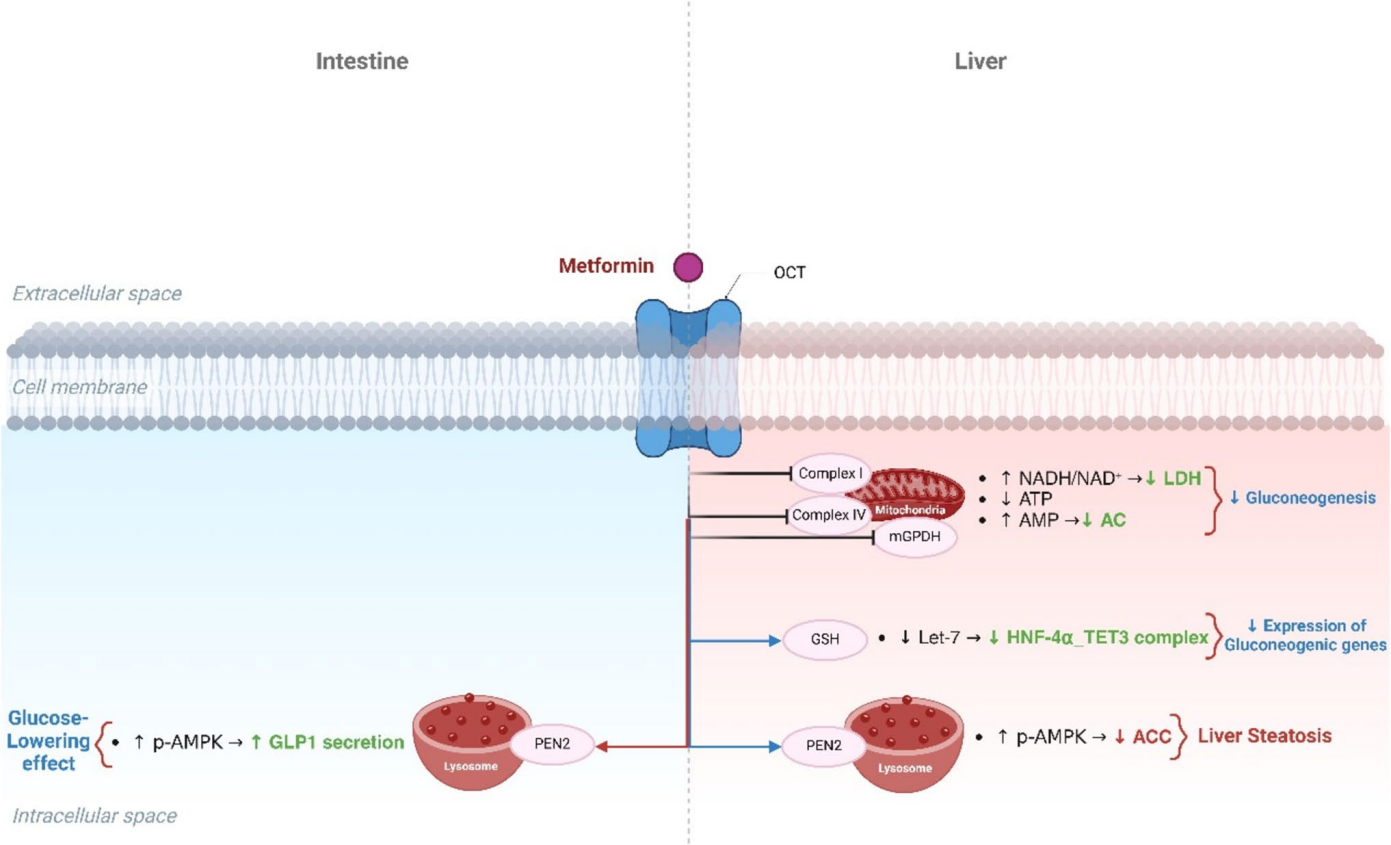
The mechanism of metformin: Metformin activates AMP-activated protein kinase (AMPK) in the liver, which leads to suppression of fatty acid synthesis and gluconeogenesis. Metformin also activates AMPK in skeletal muscle, increasing the translocation of glucose transporter 4 to the cell membrane and thereby increasing glucose uptake. Increased AMPK also inhibits mitochondrial glycerol-3-phosphate dehydrogenase (mGPD), leading to an increase in cytosolic NADH, which both stimulates the conversion of pyruvate to lactate and decreases gluconeogenesis. SREBP-1c, sterol regulatory element binding protein-1c; PEPCK, phosphoenolpyruvate carboxykinase; GAPase, glucose 6-phosphatase; GLUT 4, glucose transporter 4

Furthermore, metformin inhibits different enzymes, such as fructose 1,6-bisphosphatase 1 (FBP1) and adenylate cyclase, which are involved in gluconeogenesis by increasing AMP in the hepatocytes [27]. By increasing the AMP/ATP ratio, metformin promotes the activation of AMPK [27]. Metformin also inhibits mitochondrial glycerol phosphate dehydrogenase (mGPDH), increasing the NADH:NAD ratio and reducing the gluconeogenesis process from lactate [31]. Also, metformin increases the hepatic redox state by regulating the expression of glutathione (GSH) and oxidized glutathione (GSSG), resulting in the inhibition of many genes involved in the gluconeogenesis process [32]. Metformin, by inhibiting mitochondrial respiratory chain complex IV, indirectly suppresses the activity of mGPDH. Metformin can affect the lysosomal action by inhibiting v-ATPase independent of the AMPK pathway [33]. Besides, metformin activates liver kinase B1 (LKB1), induces the release of glucagon-like peptide 1 (GLP1) from the intestines, and regulates different genes involved in glucose homeostasis [34]. Therefore, the mechanism of metformin’s action is complex and related to other signaling pathways (Fig. 1).

Moreover, metformin reduces cellular energy by inhibiting mitochondrial oxidative phosphorylation with subsequent inhibition of gluconeogenesis, depending on the cellular ATP [35]. Metformin modulates gut microbiota and attenuates T2D-induced gut dysbiosis [36]. The regulation of gut microbiota may mediate the glucose-lowering effect of metformin [37]. It has been shown that depletion of gut microbiota attenuates the glucose-lowering effect of metformin [38]. Supporting this finding, restoration of gut microbiota by microbiota transplant improves the glucose-lowering effect of metformin [39, 40]. Metformin-induced alteration of gut microbiota induces the expression of different genes involved in regulating blood glucose, such as the *GLP1* gene [40]. It has been shown that metformin activates the release of the short-chain fatty acids (SCFAs) from gut microbiota that induce the expression of the *GLP1* gene in the enteroendocrine L cells with subsequent release of GLP1 hormone [41]. Modulation of gut microbiota by metformin enhances the synthesis and release of bile acids, improving glucose homeostasis by inhibiting intestinal farnesoid X receptor (FXR) through an AMPK-independent mechanism [42]. Moreover, metformin improves the expression of the *GDF15* gene, enhancing insulin sensitivity and having anti-inflammatory effects [43]. The central effect of metformin, mainly the anorexigenic effect, is mediated by the activation of GDF15 [44]. Therefore, through activation of AMPK, GDF15, and other signaling pathways, metformin may affect the neuropathology of ischemic stroke.

### Ischemic Stroke: An Overview

Ischemic stroke is defined as a focal neurological deficit caused by embolism or thrombosis of cerebral vessels of particular brain areas [45]. Ischemic stroke represents about 87% of total stroke type [46]. Ischemic stroke is the foremost cause of long-lasting disability and death internationally [47]. Diverse risk factors are concerned with the progress of ischemic stroke, including non-modifiable and modifiable risk factors [48–53]. Initial diagnosis of ischemic stroke is crucial as the thrombolytic treatments lead to noteworthy enhancement when administered earlier, after the onset of ischemic stroke [54]. Rising evidence from different studies demonstrated that ischemic stroke is linked with the development of oxidative stress and neuroinflammation due to unrestrained activation of inflammatory signaling pathways and the generation of reactive oxygen species (ROS) [55]. These variations may influence the development of neurodegeneration and the progression of post-stroke seizure and neurodegenerative diseases [56].

Ischemic stroke and related brain hypoxia disturb mitochondrial function, tricarboxylic acid cycle, and glycolysis, with subsequent disruption of the neuronal bioenergetics process [57]. It has been revealed that ATP exhaustion after ischemic stroke promotes the molecular and deleterious cellular events recognized as the ischemic cascade [58]. Furthermore, the absence of ATP within a few minutes before ischemic stroke activates irregular ionic efflux across the neuronal membrane [58]. It has been shown that low ATP deregulation of neuronal membrane Na +/K + ATPase decreases glutamate uptake [59]. In addition, impairment of glutamate clearance through glutamate transporters located in the neurons and glial cells is developed due to the inhibition of Na^+^/K^+^ ATPase, resulting in glutamate accumulation and neuronal excitotoxicity development [59].

Furthermore, overactivation of postsynaptic glutamate receptors by glutamate in the synaptic cleft activates calcium overload by stimulating voltage-gated calcium channels. It activates many enzymes, such as nuclease and protease [60]. Besides, cytoplasmic calcium overload through induction of mitochondrial dysfunction augments the generation of oxidant molecules, which prompt the release of nitric oxide and peroxidation of neuronal cardiolipin [61].

**Fig. 2:**
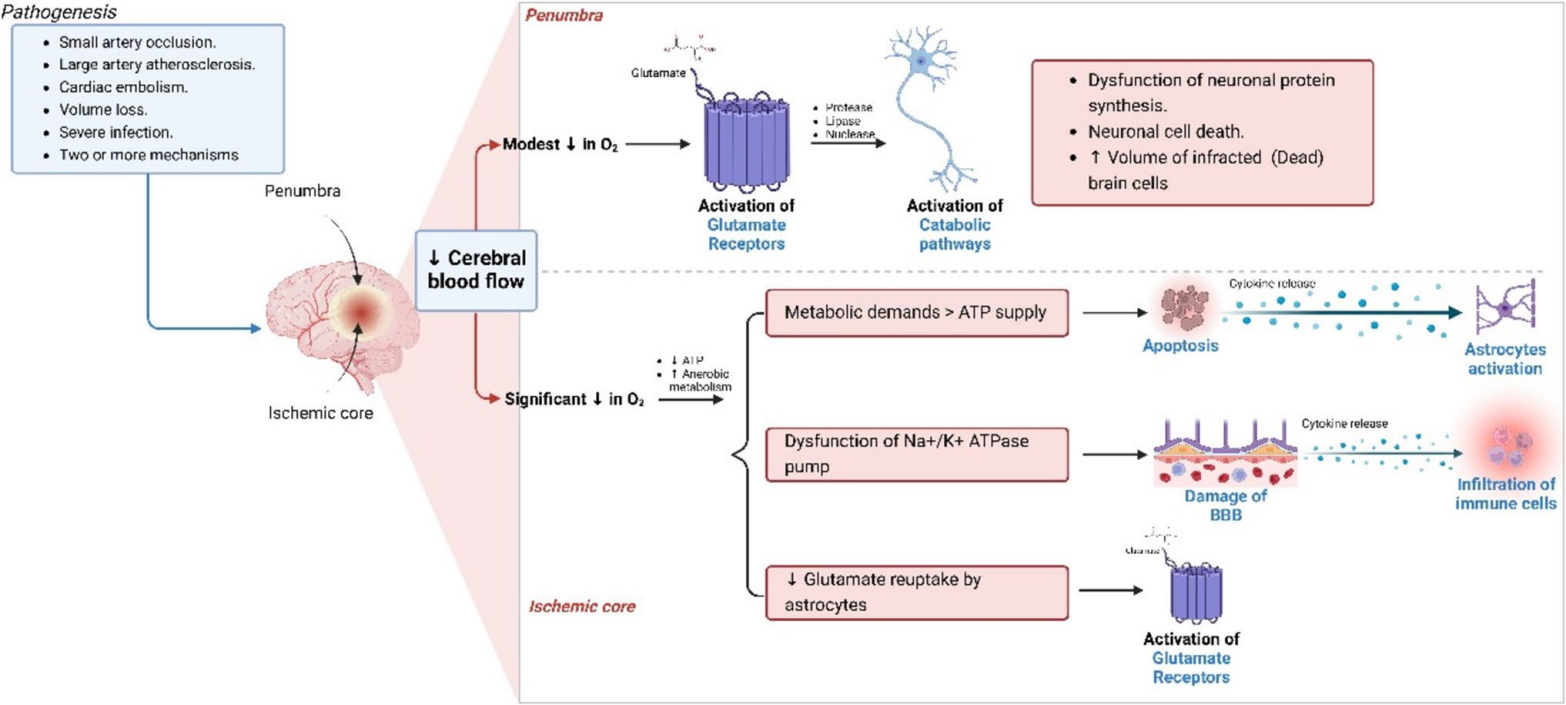
The pathogenesis of ischemic stroke: Ischemic stroke and brain hypoxia interrupt the neuronal bioenergetics process. Absence of ATP in ischemic stroke activates irregular ionic efflux across the neuronal membrane, causing abnormal neuronal membrane Na +/K + ATPase, decreases glutamate uptake, impairment of glutamate clearance resulting in the accumulation of glutamate, and the development of excitotoxicity. Overacting postsynaptic glutamate receptors by stimulating voltage-gated calcium channels initiates many enzymes, such as nuclease and protease enzymes. Cytoplasmic calcium overload through the initiation of mitochondrial dysfunction augments the generation of oxidant molecules, which prompt the release of nitric oxide and peroxidation of neuronal cardiolipin. A decrease in neuronal ATP lessens the production of intracellular antioxidant enzymes, with subsequent exaggeration of neuronal injury induced by ischemic-hypoxic changes in ischemic stroke. Brain ischemia activates neuronal PLA2 (phospholipase A2), which prompts hydrolysis of membrane phospholipid and the release of free fatty acids and lysophospholipids, which induce the development of the pro-inflammatory process in ischemic stroke

Similarly, a decrease in neuronal ATP reduces the production of intracellular antioxidant enzymes, with subsequent exaggeration of neuronal injury induced by ischemic-hypoxic changes in ischemic stroke [62]. Also, brain ischemia and ischemic reperfusion injury trigger the activation of neuronal phospholipase A2 (PLA2), which prompts hydrolysis of membrane phospholipid and the release of free fatty acids and lysophospholipids, which induce the development of the pro-inflammatory process in ischemic stroke [63]. Henceforth, the pathogenesis of ischemic stroke is complex and connected to the release of pro-inflammatory cytokines and the progression of oxidative stress **(**Fig. 2**)**.

### The Role of Metformin in Ischemic Stroke

It has been suggested that metformin could be effective against the development and progression of ischemic stroke and neurodegenerative diseases by regulating the functional activity of the brain neurovascular unit (NVU) [8, 64–66]. Metformin affects various NVU components, including pericytes, astrocytes, microglia, and vascular endothelial cells, mainly serving to protect the BBB [66]. Regulating the inflammatory response in NVU may be the primary mechanism of metformin in improving CNS-related diseases. Thus, metformin may be a potential drug for treating diseases associated with NVU deterioration. Mounting evidence from preclinical studies highlighted the protective role of metformin against the development of ischemic stroke. The administration of 50 mg/kg/day of metformin for 24 h following middle cerebral artery occlusion for 3 weeks improved neurogenesis and reduced ischemic stroke severity in mice [67]. However, in AMPK α−2 knockout mice, metformin treatment has no beneficial effects on recovery and angiogenesis, suggesting that metformin-induced angiogenic effects are mediated by AMPK [67]. Studies indicated that increasing cortical neurogenesis correlates with functional recovery following ischemic stroke [68, 69]. Angiogenesis stimulation generates new vessels to improve collateral circulation. Intrinsic genetic mechanisms and growth factors control neurogenesis. The leading process of the migrating neural progenitor cells (NPCs) is closely associated with blood vessels, suggesting that this interaction provides directional guidance to the NPCs. These findings suggest that blood vessels play an important role as a scaffold for NPC migration toward the damaged brain region [68, 69]. It has been demonstrated that angiogenesis only occurred transiently in the cortex of the ischemic hemisphere, implying that the new vessels were merely part of the clean-up after stroke rather than a contribution to neurorestoration [68, 69]. Also, induction of neurogenesis in the ischemic stroke penumbra region plays a role in eliminating cellular debris after ischemic stroke [70]. Metformin improves neuronal repair after ischemic stroke by increasing the differentiation of neural precursor cells into neuronal cells in mouse ischemic models [71, 72]. Metformin treatment also meaningfully reduced the infarct volume and alleviated functional dysfunction after stroke. Mechanistically, metformin promoted NPC migration via upregulating the *CDC42* gene expression [68]. Therefore, metformin represents an optimal candidate agent for neural repair capable of expanding the adult NPC population and subsequently driving them toward the destination for neuronal differentiation.

Moreover, metformin reduces the severity of cerebral ischemic reperfusion injury by inhibiting mitochondrial dysfunction, oxidative stress, neuroinflammation, impairment of BBB, and neuronal apoptosis [14, 65, 72–76]. Zeng et al. [77] found that metformin protects against cerebral ischemic-reperfusion injury by regulating the expression of long non-coding RNA and Rho-associated protein kinase 2 (ROCK 2), which has a neurodetrimental impact on the pathogenesis of ischemic stroke [78, 79]. Metformin can significantly alleviate acute and chronic cerebral ischemic reperfusion injury, and it has a strong regulatory effect on stroke-induced oxidative stress. The expression of lncRNA-H19 and ROCK2 could be downregulated with metformin in vivo and in vitro [77]. Thus, metformin exerts neuroprotective effects by regulating ischemic stroke-induced oxidative stress injury via the lncRNA-H19/miR-148a-3p/ROCK2 axis. These results provide new evidence that metformin may represent a potential treatment for stroke-related brain injury. In ischemic stroke, the AMPK signaling is downregulated, leading to microglia polarization and neuronal loss [11, 80]. Thus, through activating AMPK signaling, metformin reduces ischemic stroke severity in the experimental stroke models [81, 82]. It has been shown that metformin upregulated the brain-derived neurotrophic factor (BDNF) expression and increased phosphorylation levels of AMPK and cAMP-response element binding protein (CREB) in the ischemia penumbra. This effect of metformin is reversed by Compound C [81, 82]. Therefore, metformin improves BDNF expression in the cerebral ischemic penumbra via the activation of the AMPK/CREB pathway, thereby protecting cerebral ischemic reperfusion injury.

Furthermore, metformin at 20 mg/kg improved neurological function and attenuated brain edema, oxidative stress, and BBB permeability disruption 24 h after subarachnoid hemorrhage, promoted mitophagy in an AMPK-dependent manner. Metformin attenuated early brain injury after subarachnoid hemorrhage in rats through AMPK-dependent signaling [81, 82]. These protective effects might be achieved by regulating mitochondrial morphology and promoting mitophagy [64, 65]. Furthermore, due to its anti-inflammatory and antioxidant properties, metformin attenuates the pathogenesis of ischemic stroke in animal models [81, 83]. Metformin, by promoting the expression of neuroprotective BDNF and antioxidant genes, inhibits the expression of inflammatory signaling pathways [81, 83]. Moreover, metformin encourages the activation of AKT1 and reduces the phosphorylation of JNK3 and c-Jun and elevation of cleaved caspase-3 in ischemia/reperfusion brains [14, 74]. PI3 K inhibitor reversed all the protective effects [81, 82], indicating that metformin protects the hippocampus from ischemic damage through PI3 K/Akt1/JNK3/c-Jun signaling pathway [84]. A meta-analysis of preclinical studies highlighted that administration of metformin attenuates the pathogenesis of ischemic stroke and improves the functional recovery of neurological deficits after ischemic stroke [84]. Notably, metformin treatment plays a neuroprotective role and improves pathological and behavioral outcomes in rodent stroke models [76, 85]. Although multiple limitations regarding animal study methodology exist, the results of different studies may provide an essential situation for future preclinical and clinical studies on stroke outcome recovery [81, 82].

In clinical settings, many studies highlighted the neuroprotective role of metformin against ischemic stroke. A double-blind, placebo-controlled clinical trial illustrated that treatment with metformin 500 mg/twice daily in non-diabetic patients for 7 days following ischemic stroke decreased ischemic stroke severity and improved neurological deficits after 3 months [86]. Therefore, administration of metformin in T2D patients before stroke onset may be associated with reduced neurological severity and improved acute-phase therapy outcomes. A cohort study illustrated that pre-ischemic stroke use of metformin was linked with favorable clinical outcomes following the development of ischemic stroke in T2D patients [87]. A retrospective cohort study confirmed that using metformin in pre-ischemic stroke that continued during the post-ischemic stroke period had a potential protective effect and improved the clinical outcomes at 90 days [88]. However, a prospective study highlighted that metformin use did not alleviate the clinical outcomes and the severity of ischemic stroke in T2D patients [89], though this small sample study may not give a complete picture regarding the therapeutic efficacy of metformin on ischemic stroke. A longitudinal study conducted by Cheng et al. [90] indicated that follow-up of T2D patients on metformin therapy reduced the risk of ischemic stroke compared to non-metformin use, suggesting a preventive role against the development of ischemic stroke in T2D patients. Likewise, a retrospective study disclosed that T2D patients on metformin therapy who received thrombolytic drugs after acute ischemic stroke had better functional recovery at 3 months [91]. A multicenter, double-blind clinical trial showed that metformin was safe and well tolerated in the prevention of secondary stroke in patients with transient ischemic attack [92]. An updated systematic review and meta-analysis involving 1051 studies of 11,589 T2D patients showed that pre-ischemic stroke use of metformin improves clinical outcomes and reduces mortality in T2D patients with ischemic stroke [93]. These findings indicated that metformin has a neuroprotective effect against the development and progression of ischemic stroke.

On the other hand, many preclinical studies showed that acute metformin treatment may exacerbate the neuropathology of ischemic stroke by increasing neuronal lactate [94]. AMPK is highly expressed in cortical and hippocampal neurons under normal and ischemic conditions. AMPK activity is increased following both middle cerebral artery occlusion and oxygen–glucose deprivation. Pharmacological inhibition of AMPK by either C75, a known modulator of neuronal ATP levels, or compound C reduced stroke damage. In contrast, activation of AMPK by 5-aminoimidazole-4-carboxamide ribonucleoside exacerbated damage. Mice deficient in neuronal nitric-oxide synthase decreased stroke damage and AMPK activation compared with wild type, suggesting a possible interaction between NO and AMPK activation in stroke [94]. These findings demonstrate a role for AMPK in the response of neurons during metabolic stress and suggest that in ischemia, the activation of AMPK is deleterious.

High lactate levels and stimulation of endothelial NO by metformin induce more neuronal injury in the ischemic stroke model [95]. AMPK activation has been described as one of the pharmacological mechanisms that explain metformin’s action and leads to neuroprotective effects [9]. Most experiments done in the cerebral ischemia model via middle cerebral artery occlusion in rodents had positive results favoring metformin’s neuroprotective role. They involved several cellular pathways like oxidative stress, endothelial NO synthase activation, activation of angiogenesis and neurogenesis, autophagy, and apoptosis. Acute AMPK activation at the neuronal level produces harmful effects with an increased cerebral infarct area compared with chronic treatments for at least 2 to 3 weeks before ischemia in experimental models. Chronic therapy with metformin reduces AMPK activation and may produce similar effects as in studies on preconditioning before ischemia [95].

**Fig. 3:**
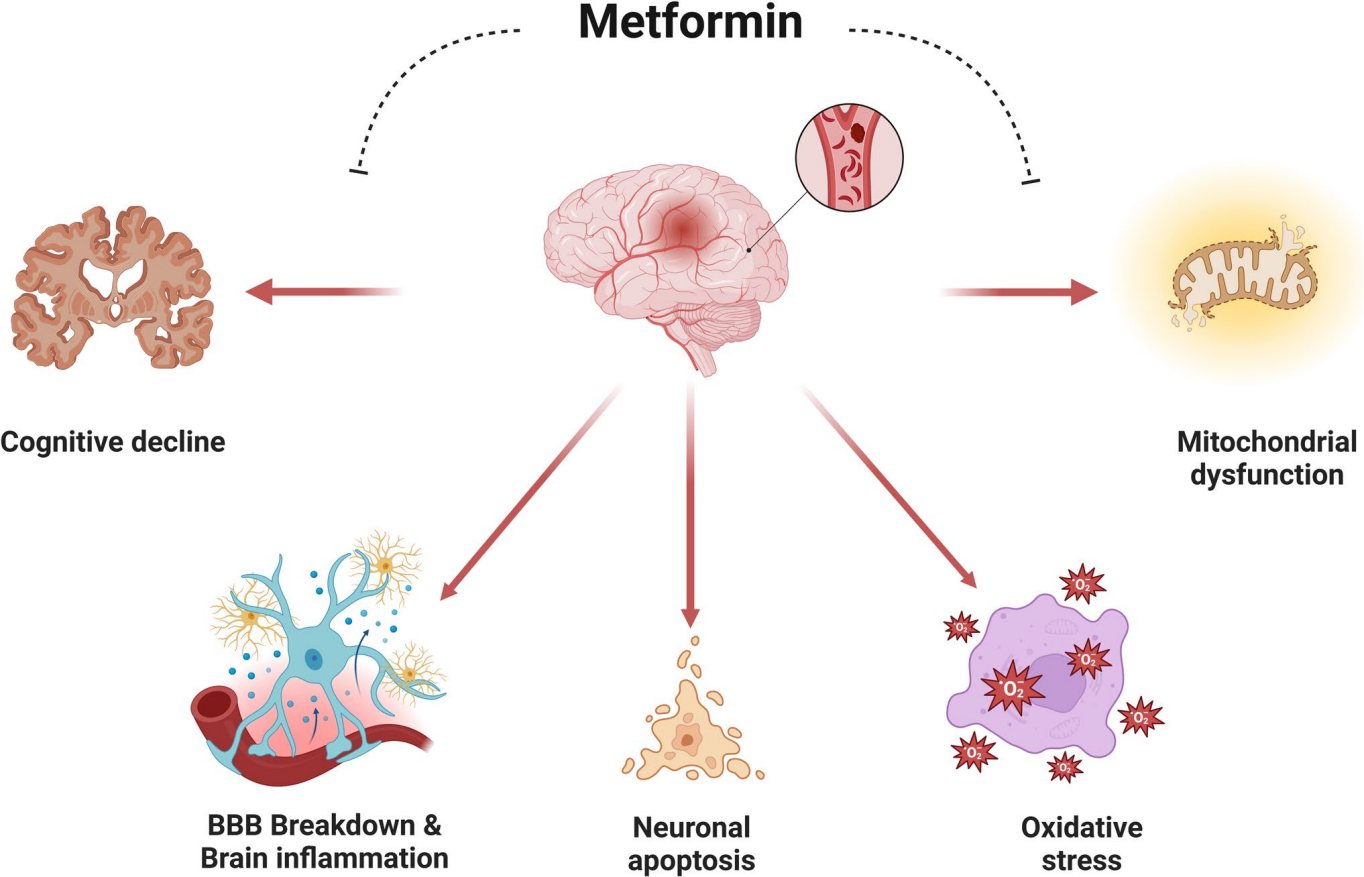
Role of metformin in ischemic stroke: Metformin, by regulating mitochondrial dysfunction, oxidative stress, neuronal apoptosis, BBB permeability, and inflammation, improves cognitive impairment in ischemic stroke

Furthermore, metformin activates astrocytic glycolysis, causing lactic acidosis [10]. Glucose transported to the brain is metabolized to lactate in astrocytes and supplied to neuronal cells via a monocarboxylic acid transporter (MCT). Lactate is used in neuronal cells for various functions, including learning and memory formation. It has been shown that lactate can block stroke-induced neurodegeneration. Under physiological conditions, astrocytes’ lactate production and release are regulated by lactate dehydrogenase (LDH) changes and MCT expression [96]. Lactate production and supply are regulated through hypoxia-inducible factor (HIF)−1α expression, especially with hypoxic stimulation, which may promote neuronal apoptosis; though, neuronal survival may be promoted via HIF-1α. Stroke stimulation could prevent neurodegeneration by substantially enhancing lactate production and upregulating MCT4 expression to accelerate lactate supply. However, studies using astrocytes derived from animal stroke models revealed significantly reduced lactate production and MCT expression [96]. These findings suggest that the lack of lactate supply may strongly contribute to hypoxia-induced neurodegeneration. As well, reduced lactate supply from astrocytes could facilitate stroke-induced neurodegeneration. Therefore, astrocyte-derived lactate may contribute to stroke prevention [96]. However, metformin prevents neuronal loss in the striatum following cerebral ischemic reperfusion injury due to neuronal energy depletion [97]. Nonetheless, these findings are not entirely translated into clinical settings. Indeed, metformin has a neuroprotective role in preventing and treating ischemic stroke **(**Fig. 3**)**. However, the precise underlying mechanism for the neuroprotective effect is not fully elucidated.

### Metformin and AMPK in Ischemic Stroke

**Fig. 4:**
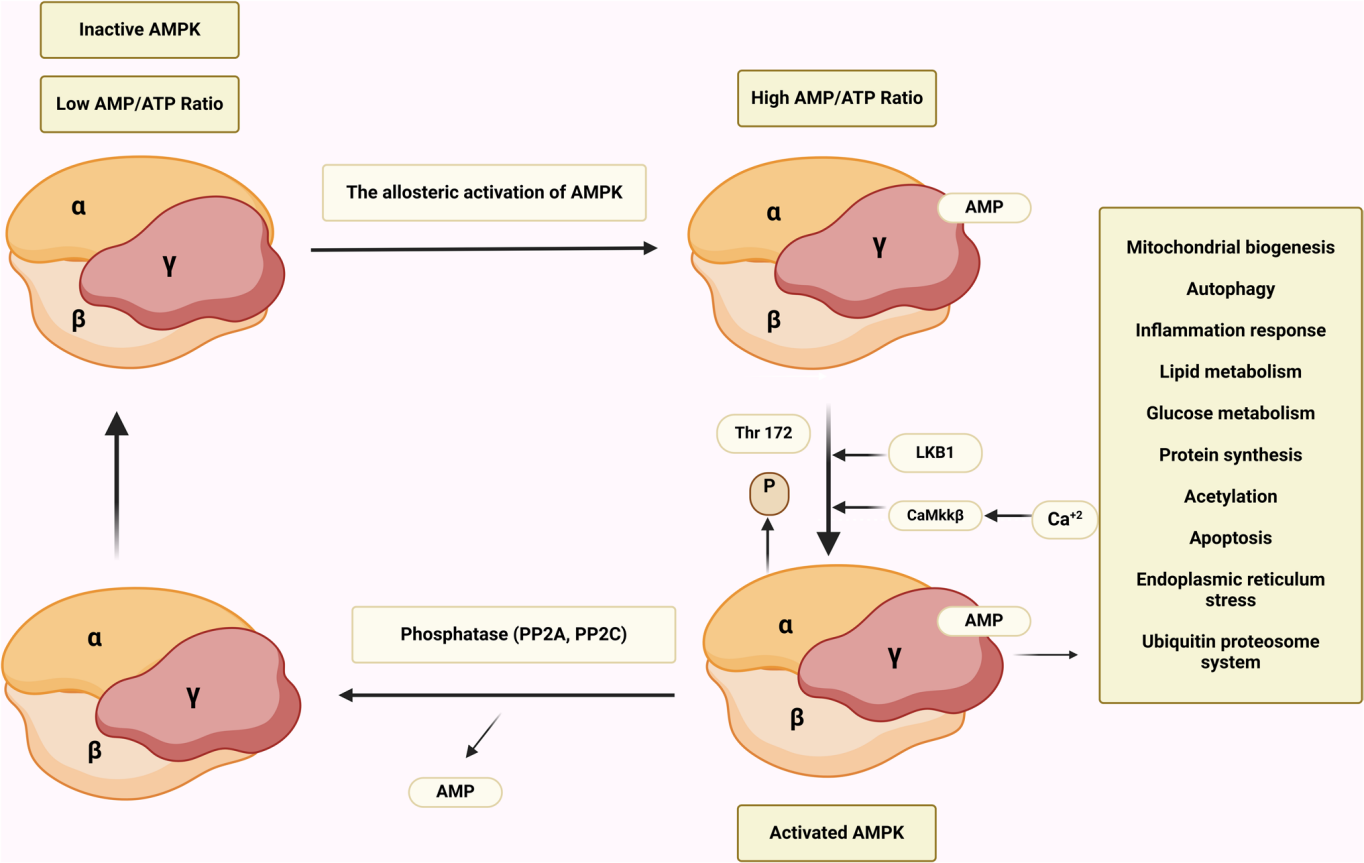
Mechanistic effects of AMPK in ischemic stroke: AMPK by modulating oxidative stress increases LC-3II, which activates neuronal autophagy, resulting in inhibition of neuronal apoptosis with suppression of mitochondrial dysfunction. By this mechanism and direct inhibition of glutamate excitotoxicity and neuroinflammation, AMPK attenuates oxidative stress in ischemic stroke

AMPK is a conserved serine/threonine kinase, a peripheral energy sensor. It was first extracted in 1994 by Carling et al. [98]. AMPK is critical in regulating energy under stress and physical exercise in different organs such as the brain, kidney, heart, liver, pancreas, and lungs [99]. AMPK resists the development of brain ischemia by regulating the catabolic pathway and ATP consumption [100]. AMPK is a complex protein with three subunits: α, β, and γ. The α subunit consists of the α carboxy-terminal domain (α-CTD) and the auto-inhibitory domain (AID), which reduces the AMPK activity. The β subunit consists of a carbohydrate-binding module (CBM) and β-CTD. The CBM inhibits AMPK activity in the presence of excess glycogen. The γ subunit comprised of NTR and cystathionine β synthase (CBS) that increase the activation of AMPK activity [101]. The AMPK is involved in various cellular processes such as mitochondrial biogenesis, autophagy, inflammatory response, and other cellular function (Fig. 4).

The neurons are poor for glycogen storage and mainly utilize glucose, making them vulnerable to injury following ischemic stroke [102, 103]. Under stressful conditions, AMPK is activated in ischemic stroke to maintain neuronal homeostasis and activity. Different studies highlighted that AMPK has a neuroprotective effect against ischemic stroke [102, 103]. Pre-treatment with an AMPK activator, metformin, attenuates the inflammatory and ischemic stress disorders in rat ischemic stroke. However, pre-treatment with AMPK inhibitor reduces the protective effect of metformin in the ischemic stroke model [102]. Likewise, pre-treatment with metformin 200 mg/kg/day for 2 weeks reduces global cerebral ischemia and ischemic reperfusion injury by activating the AMPK signaling. However, inhibition of AMPK by compound C reverses this protective effect [103]. These findings suggest that metformin prevents the progression of ischemic stroke by regulating mitochondrial biogenesis and inhibiting oxidative and inflammatory disorders through an AMPK-dependent pathway.

**Fig. 5:**
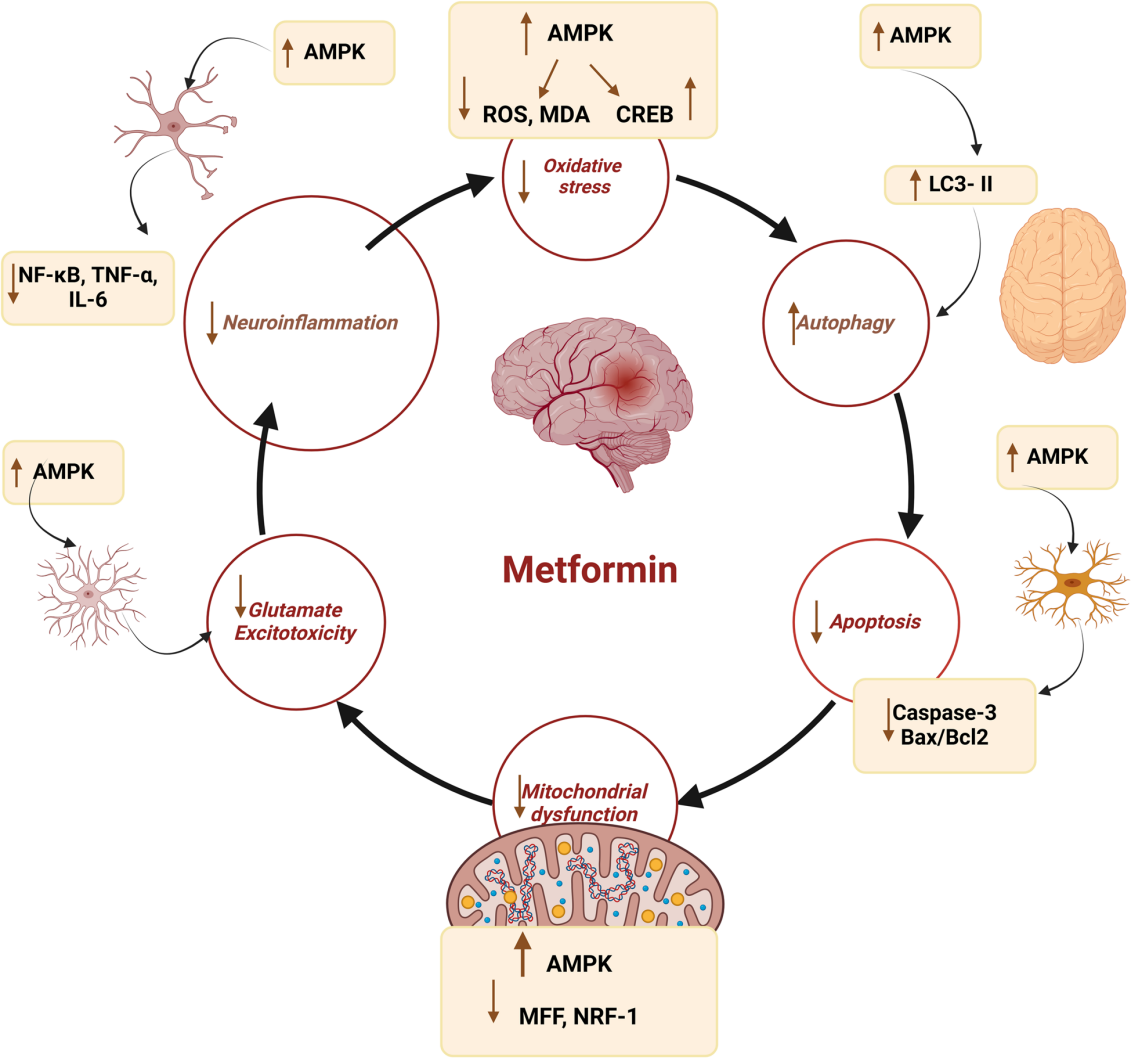
The neuroprotective effect of metformin in ischemic stroke: Activation of AMPK by allosteric activators improves the expression of many signaling pathways that regulate neuronal biogenesis by increasing neuronal glucose metabolism and inhibiting oxidative stress and inflammation in ischemic stroke

Furthermore, a natural product such as sinomenine, which has anti-inflammatory and anti-apoptotic effects, is effective against experimental ischemic stroke by inhibiting neuroinflammation via AMPK activation [104–106]. In vitro and in vivo findings showed that administration of the peptide hormone apelin 13 decreases the progression of ischemic stroke induced by ischemic reperfusion injury by activating AMPK signaling, which inhibits oxidative stress and related inflammation [107]. A natural product, berberine, has also been established to regulate microglial polarization and accelerated angiogenesis after experimental ischemic stroke via activation of AMPK signaling [108]. Chen et al. [109] illustrated that glycine reduces oxygen–glucose deprivation in the ischemic model of PC12 derived from patients with ischemic stroke by inducing AMPK signaling. Inhibition of AMPK by miR-27b in cultured neural stem cells under oxygen–glucose deprivation reduces neuronal proliferation. Administration of miR-27b inhibitor in a mouse ischemic stroke model improves spatial memory and functional recovery by increasing AMPK activity [110]. These findings indicated that AMPK has a neuroprotective effect against the development and progression of ischemic stroke (Fig. 5).

However, metformin failed to produce a noteworthy effect against acute ischemic stroke in animals and humans [86, 111]. Metformin can improve the neurological function and oxidative stress status of acute stroke patients with T2D through the AMPK/mTOR signaling pathway. The underlying cause for this failure is the reduced ability of metformin to cross the BBB [112], which needs hours to reach the intracellular concentration to activate AMPK [113]. Interestingly, NO synthase (NOs) is critical in mediating the activation of AMPK in ischemic stroke. Therefore, deficient mice experience low neuronal injury following ischemic-reperfusion injury [94]. It has been shown that AMPK activates endothelial NOs and hypoxia-inducible factor 1 (HIF-1), which affects neuronal survival in ischemic stroke [114, 115]. Therefore, persistent AMPK activation in response to hypoxia, oxidative stress, and inflammation reduced the energy balance of compromised neurons in ischemic stroke. AMPK increases stress in the compromised neurons by inducing survival in an energy-deficient environment in ischemic stroke until the restoration of blood flow in the ischemic area [94]. Thus, AMPK may be detrimental in the early phase of ischemic stroke by inducing neuronal apoptosis.

Furthermore, findings from preclinical studies illustrated that AMPK activation triggers neuronal apoptosis in human neuroblastoma cells [116] and promotes apoptosis in mouse neuroblastoma cells via induction of inflammation and oxidative stress [117]. Moreover, the neurotoxicity of an environmental pollutant, tributyltin, is mediated by activating AMPK, which is attenuated by AMPK inhibitor compound C [118]. However, the in vitro cell response differs from the in vivo effect of AMPK since the dynamic response of neurons in conjunction with microglia to the impact of AMPK is sequential and time-dependent. AMPK inhibition by C75 mitigates neuronal injury in experimental ischemic stroke [119]. Deletion of AMPKα2 reduces the protective effect of compound C in the ischemic stroke model [120]. Therefore, over-activation of AMPK under energy deficiency in ischemic stroke may exacerbate neuronal injury and trigger the progression of neuronal apoptosis.

Hence, AMPK activation before the development of ischemic stroke enhances neuronal survival, though over-activated AMPK induces neuronal apoptosis. For example, activating AMPK in response to glutamate excitotoxicity and hypoxia increases neuronal tolerance to ischemia and prevents neuronal injury [121]. Therefore, substantial controversy exists regarding the potential role of AMPK signaling in ischemic stroke. Herein, metformin’s role in ischemic stroke through activation of AMPK needs further research.

### Metformin and GDF15 in Ischemic Stroke

GDF15 is a member of the TGF-β superfamily; it was discovered more than 25 years ago as a macrophage inhibitory cytokine 1. GDF15 consists of two exons and one intron in the pre-prodomain [122]. GDF15 is highly expressed in the placenta and prostate, though it can be detected in other organs such as the heart, kidney, and lung [123, 124]. Circulating GDF15 level is low in healthy humans but tends to increase with age without sex difference. Its level is augmented by exercise and stress. GDF15 is mainly secreted from skeletal muscles during exercise [124]. It was suggested that GDF15 acts by activating TGF-β receptor 2 (TGF-βR2), though further studies did not confirm this. In particular, GDF15 activates glial-derived neurotrophic factor receptor alpha-like (GFRAL), which is expressed in the brain, mainly in the area postrema. The GFRAL needs co-receptor RET, which GDF15 also activates. The anorexigenic of GDF15 is mediated via the activation of GFRAL [125]. GDF15 reduces body weight, improves peripheral insulin sensitivity, and has a potent anti-inflammatory effect [85]. Therefore, the GDF15/GFRAL axis is a metabolic pathway to control food intake. GDF15 is viewed as a stress cytokine released in response to tissue injury, such as acute liver injury and cardiovascular injury. Metformin has been shown to activate the expression and release of GDF15 [6]. In primary mouse hepatocytes, metformin stimulates the secretion of GDF15 by increasing the expression of activating transcription factor 4 (ATF4) and C/EBP homologous protein (CHOP; also known as DDIT3) in mice. Increased serum GDF15 is also associated with weight loss in T2D patients treated with metformin [6]. Metformin inhibits the feeding center by activating the release of GDF15 in mice with a high-fat diet [43]. Supporting this finding, administration of GFRAL antagonist reduces metformin’s anorexigenic and weight-lowering properties [43]. Therefore, prolonged use of metformin is linked with increasing the circulating level of GDF15 in a dose and time-dependent manner [126]. In addition, metformin-mediated GDF15 activation could be a mechanistic pathway that controls energy balance and metabolic effects [127]. Remarkably, GDF15 circulating level is correlated with diabetic complications such as diabetic retinopathy and diabetic neuropathy as a compensatory mechanism to reduce the inflammatory and oxidative stress disorders in T2D [128].

Furthermore, GDF15 circulating level has been shown as a robust prognostic biomarker of mortality in patients with ischemic stroke [12]. Preclinical findings showed that cortical GDF15 overexpression is correlated with cerebral injury in animal models of ischemic stroke [129, 130]. GDF-15 is a potent neurotrophic factor for lesioned dopaminergic neurons in the substantia nigra, and GDF-15-deficient mice show progressive postnatal losses of motor and sensory neurons. In MCAO, GDF-15 mRNA expression is augmented in the hippocampus and parietal cortex at 3 h and 24 h after the lesion. Moderate induction of GDF-15 immunoreactivity has been observed in a small number of microglial cells after MCAO [129, 130]. Therefore, GDF-15 orchestrates post-lesional responses other than the trophic support of neurons in ischemic stroke. In the brains of rats and mice, where GDF-15 is physiologically expressed in the choroid plexus epithelium, cortical lesioning or cerebral ischemia has been shown to induce a significant increase of GDF-15 expression in the injured cortex [131]. In pathological conditions, GDF-15 may originate from several tissues, particularly inflammatory cells. However, it has been shown that cryogenic cerebral lesions induced GDF-15 expression in macrophages, neurons, and microglial cells [131].

In clinical settings, a prospective study on 173 patients illustrated that the plasma baseline level of GDF15 at admission was correlated with 3-month mortality [12]. In addition, a plasma baseline level of GDF15 predicts ischemic stroke risk in hypertensive patients [132] and ischemic stroke risk in patients with atrial fibrillation [133]. Furthermore, the plasma baseline level of GDF15 is correlated with subclinical brain damage and incident ischemic stroke [134]. As well, the circulating level of GDF15 is associated with the risk of depression following ischemic stroke [135]. Yang et al. [136] found that a high circulating level of GDF15 in T2D patients is correlated with the risk of ischemic stroke.

Remarkably, increasing the circulating level of GDF15 may reduce the risk of post-stroke cognitive impairment [136]. GDF15 promotes angiogenesis and inhibits apoptosis by reducing inflammatory reactions in neurological disorders [137]. Besides, GDF15 suppresses NF-κB activation and facilitates the generation of pro-inflammatory cytokines, such as TNF-α and MCP-1 [138], associated with the pathogenesis of ischemic stroke. The sources of GDF15 during ischemic stroke may be from inflammatory cells, mainly macrophages and microglia [129]. It has been shown that experimental ischemic stroke induces the expression of GDF15 mRNA in the ipsilateral parietal cortex and hippocampus [130], suggesting that increasing GDF15 expression could be a compensatory mechanism to mitigate the inflammatory process in ischemic stroke. Supporting this claim, the upregulation of GDF15 in the neurons after ischemic stroke plays a role in orchestrating post-lesional response and trophic effects on the neurons [130]. Therefore, these findings indicated that metformin-induced expression of GDF15 could be a mechanistic pathway against the development and progression of ischemic stroke.

### The Interaction Between GDF15 and AMPK

It has been illustrated that a low metformin dose increases the expression of GDF15 mRNA through an AMPK-dependent pathway in cultured hepatocytes and myotubes. In addition, the upregulation of GDF15 is essential for metformin to activate AMPK [139]. Cultured hepatocytes and myotubes treated with metformin showed AMPK-mediated increases in GDF15 levels independently of its central receptor, GFRAL. At the same time, GDF15 knockdown blunted the effect of metformin on AMPK activation, suggesting that AMPK is required for the metformin-mediated increase in GDF15, which in turn is needed to sustain the full activation of this kinase independently of the CNS [139]. Additionally, knockdown of the AMPKα subunit in primary hepatocytes and myotubes prevented the increase in GDF15 levels caused by metformin, suggesting that this drug increases GDF15 levels through AMPK [139]. Furthermore, activation of AMPK by A769662 increased hepatic GDF15 levels [140]. Pharmacological activation of AMPK β1-containing complexes consistently induced GDF15 levels, whereas liver *Gdf15* gene expression and serum GDF15 levels were attenuated in mice lacking AMPK β1-containing complexes [140]. Similarly, agonists of PPARβ/δ increase GDF15 by activating AMPK via the AMPK-p53 axis [33]. Furthermore, the p-AMPK levels are reduced in the liver and skeletal muscle of mice lacking the *GDF15* gene, confirming the activation of AMPK by GDF15 [139]. These findings suggest a positive feedback loop between GDF15 and AMPK in metformin. AMPK, under stressful conditions, also promotes the expression of GDF15 to preserve energetic balance [140]. Direct activation of AMPK β1-containing complexes by A769662 increased hepatic GDF15 expression, circulating GDF15, and suppressed food intake, independent of ER stress [140]. Moreover, the metabolic effect of GDF15 is mediated by activating AMPK signalling in the peripheral organs, such as skeletal muscles [141]. Pharmacological PPARβ/δ activation increases GDF15 levels, ameliorates glucose intolerance, fatty acid oxidation, ER stress, and inflammation, and activates AMPK in HFD-fed mice. In contrast, these effects are abrogated by injecting a GDF15 neutralizing antibody and in GDF15 knockout mice. The AMPK-p53 pathway is involved in the PPARβ/δ-mediated increase in GDF15, which in turn activates again AMPK. Consistently, GDF15 knockout mice show reduced AMPK activation in skeletal muscle, whereas GDF15 administration results in AMPK activation in this organ [141]. Thus, these data reveal a mechanism by which PPARβ/δ activation increases GDF15 levels via AMPK and p53, which in turn mediates the metabolic effects of PPARβ/δ by sustaining AMPK activation. Hence, the metformin action in ischemic stroke is mediated by dual activation of AMPK and GDF15, which have anti-inflammatory and antioxidant effects.

These studies display that the timing, duration, and amount of AMPK activation are key factors in determining the final downstream effects of AMPK on the ischemic brain. The detrimental impact of acute AMPK activation may be mediated, at least in part, by enhancement of lactic acidosis. Chronic Metformin treatment may lead to sublethal metabolic stress and downregulate AMPK, protecting the brain from subsequent injury.

Taken together, metformin, through appropriate activation of the AMPK/GDF15 axis, may reduce the inflammatory and oxidative stress disorders in ischemic stroke. Metformin seems to be detrimental in the acute phase of ischemic stroke by activating AMPK/GDF15 in energy-deficient neurons. However, activating the AMPK/GDF15 axis by metformin in the chronic phase of ischemic stroke is beneficial by inhibiting inflammation, oxidative stress, BBB injury, and neuronal apoptosis, and enhancing neurogenesis.

Despite these findings, numerous limitations are present in the present review. Though we searched the enormous majority of prominent databases, there is still a scarcity in the recovered articles since only articles published in English were included, and some studies with negative results were overlooked. Consequently, the effect of metformin may be overstated. The quality of this review was limited due to some degree of heterogeneity across investigated studies. The animal model background, anesthetic drugs and route, stroke subtype, metformin administration time point, and outcome measurement time may influence the quality. Consequently, sufficient evidence is needed for the future. Furthermore, diverse intervention time spans may lead to substantial heterogeneity among the studies. Generally, significant work remains to be done for the clinical translation of metformin treatment for stroke patients. Notably, most of the findings of the present review were derived from preclinical studies, which are not completely translated into clinical settings. Thus, clinical trials and prospective studies regarding the effects of metformin in acute and chronic ischemic stroke are recommended in this regard.

## Conclusions

Metformin has neuroprotective effects against the development and progression of ischemic stroke. However, metformin may exacerbate the pathogenesis of ischemic stroke. This controversial point may be related to the impact of metformin on the different signaling pathways, such as AMPK and GDF-15. Metformin, through activation of AMPK signaling, reduces the severity of ischemic stroke. AMPK has a neuroprotective effect against ischemic stroke, though it may exacerbate neuronal injury in ischemic stroke. Therefore, there is an intense controversy regarding the potential role of AMPK signaling in ischemic stroke. Herein, metformin’s role in ischemic stroke through activation of AMPK needs further research.

Moreover, increasing the circulating level of GDF15 may reduce the risk of post-stroke cognitive impairment. Therefore, metformin-induced expression of GDF15 could be a mechanistic pathway against the development and progression of ischemic stroke.

Metformin seems to be detrimental in the acute phase of ischemic stroke by activating AMPK/GDF15 in energy-deficient neurons. Nevertheless, activating the AMPK/GDF15 axis by metformin in the chronic phase of ischemic stroke is beneficial by inhibiting inflammation, oxidative stress, BBB injury, and neuronal apoptosis, and enhancing neurogenesis. Hence, metformin activation of the GDF15 signaling pathway seems to benefit acute ischemic stroke. Conversely, activation of the AMPK signaling pathway by metformin appears to produce a beneficial effect in chronic ischemic stroke. Thus, selective activators of GDF15 and AMPK signaling pathways may effectively manage acute and chronic ischemic strokes, respectively.

Collectively, metformin, through suitable activation of the AMPK/GDF15 axis, may reduce the inflammatory and oxidative stress disorders in ischemic stroke. Therefore, additional studies are warranted to explore the exact role of metformin in acute ischemic stroke regarding the molecular effects of the AMPK/GDF15 axis.

## Data Availability

No datasets were generated or analysed during the current study.
